# Using the zebrafish lateral line to uncover novel mechanisms of action and prevention in drug-induced hair cell death

**DOI:** 10.3389/fncel.2015.00046

**Published:** 2015-02-18

**Authors:** Tamara M. Stawicki, Robert Esterberg, Dale W. Hailey, David W. Raible, Edwin W Rubel

**Affiliations:** ^1^Virginia Merrill Bloedel Hearing Research Center, University of WashingtonSeattle, WA, USA; ^2^Department of Biological Structure, University of WashingtonSeattle, WA, USA; ^3^Department of Otolaryngology, Head and Neck Surgery, University of WashingtonSeattle, WA, USA

**Keywords:** aminoglycoside, chemical genetics, cisplatin, hair cell, lateral line, mechanotransduction, ototoxicity, zebrafish

## Abstract

The majority of hearing loss and balance disorders are caused by the permanent loss of mechanosensory hair cells of the inner ear. Identification of genes and compounds that modulate susceptibility to hair cell death is frequently confounded by the difficulties of assaying for such complex phenomena in mammalian models. The zebrafish has emerged as a powerful animal model for genetic and chemical screening in many contexts. Several characteristics of the zebrafish, such as its small size and external location of mechanosensory hair cells within the lateral line sensory organ, uniquely position it as an ideal model organism for the study of hair cell toxicity. We have used this model to screen for genes and compounds that affect hair cell survival during ototoxin exposure and have identified agents that would not be expected to play a role in this process based on *a priori* knowledge of their function. The identification of such agents yields better understanding of hair cell death and holds promise to stem hearing loss and balance disorders in the human population.

## Introduction

Hair cell death is a leading cause of hearing and balance disorders in the human population. Hair cells are sensitive to multiple insults, including aging, noise and certain therapeutic drugs (Cheng et al., 2005; Konings et al., 2009; Schacht et al., 2012; Yamasoba et al., 2013). Two major classes of drugs that cause hair cell death resulting in hearing and/or vestibular deficits are aminoglycoside antibiotics (AGs) and platinum-based chemotherapeutics. There is a large degree of variability in the effects of exposure on ototoxicity. AG-induced hearing loss has been reported in up to 22% of patients (Moore et al., 1984), and vestibular impairment has been seen in up to 11% of patients (Lerner et al., 1986). Hearing loss has been reported in up to 100% of patients who have been given the anticancer agent cisplatin (Kopelman et al., 1988; McKeage, 1995).

Since the discovery of ototoxic side effects numerous studies have been conducted in an attempt to better understand the cellular mechanisms underlying drug-induced hair cell death. These studies have found that apoptotic-like cell death pathways and reactive oxygen species (ROS) production appear to play key roles in these events (Huth et al., 2011; Tabuchi et al., 2011; Schacht et al., 2012). Due to the role of ROS in drug-induced ototoxicity, a number of drugs with antioxidant or ROS scavenging capabilities are currently in clinical trials to prevent ototoxicity (Langer et al., 2013). However, as there are currently no proven effective treatments for drug-induced toxicity, there remains a need to both better understand the mechanisms behind this process as well as to identify novel protective drugs. Recent studies from our labs and others have used the zebrafish lateral line system, a superficial sensory organ comprised of mechanosensory hair cells, to attain these goals. Hair cells of the zebrafish lateral line are morphologically, functionally, and molecularly similar to mammalian hair cells (Whitfield, 2002; Nicolson, 2005). Moreover, lateral line hair cells are sensitive to the same ototoxic insults as mammalian hair cells (Harris et al., 2003; Ton and Parng, 2005; Ou et al., 2007). Critically important is that due to their fecundity, rapid development and the superficial hair cell system, it is possible to perform large-scale chemical and genetic screens in zebrafish that would not be feasible in mammals. This superficial location of lateral line hair cells also make it possible to use the zebrafish for *in vivo* imaging studies aimed at understanding the pathways responsible for the progression of hair cell death and survival. In this review we will highlight some of the recent advances in both uncovering novel cellular pathways involved in drug-induced hair cell death, as well as novel potential treatments utilizing the zebrafish lateral line system.

## Screening for genes that protect against AG toxicity

One advantage of using zebrafish as a model system is the ability to carry out forward genetic screens (Knapik, 2000; Patton and Zon, 2001). Such screens provide an unbiased approach to identify novel genes involved in a process of interest. They have been used to identify genes involved in a wide range of biological processes ranging from early development to behavior (Driever et al., 1996; Granato et al., 1996). The original goal of our project was to develop a screening system to identify genes that modulate hair cell susceptibility to ototoxic agents (Harris et al., 2003). Our lateral line screening to date has identified three zebrafish mutants that show resistance to neomycin-induced hair cell death. The disrupted genes in these mutants are *cc2d2a*, a ciliary transition zone gene (Owens et al., 2008); *slc4a1b*, a chloride/bicarbonate exchanger (Hailey et al., 2012); and *gcm2*, a transcription factor important for global pH regulation (Stawicki et al., 2014). None of these genes were previously implicated in hair cell toxicity, nor are they genes that would have been examined by a candidate approach.

Mutations in *cc2d2a* have been found in patients suffering from the ciliopathies Meckel and Joubert syndrome (Gorden et al., 2008; Tallila et al., 2008). While mammalian auditory hair cells lose their kinocilia after birth (Kikuchi and Hilding, 1965; Kimura, 1966), vestibular hair cells maintain theirs (Ernstson and Smith, 1986), and therefore CC2D2A may play a role in the vestibular toxicity of aminoglycosides in mammals. CC2D2A associates with a number of ciliopathy gene products at the transition zone of cilia. This protein complex is believed to function as a gate-keeper for proteins exiting and entering the cilia, therefore influencing cilia-dependent signaling pathways (Chih et al., 2011; Garcia-Gonzalo et al., 2011; Williams et al., 2011). As uptake of both FM1-43 and gentamicin-Texas Red is unaffected in the *cc2d2a* mutant, CC2D2A is presumably acting intracellularly in aminoglycoside toxicity (Owens et al., 2008). CC2D2A contains a C2 domain, a Ca^2+^ dependent membrane-binding domain (Nalefski and Falke, 1996). As a breakdown of normal Ca^2+^ regulation is known to play a role in AG-induced hair cell death (Hirose et al., 1999; Matsui et al., 2004; Esterberg et al., 2013, 2014) it is tempting to speculate that CC2D2A links Ca^2+^ signaling to other signaling pathways responsible for the ultimate death of the cell. However, as of yet there is no data to confirm this.

The other two genes identified as protective encode for proteins essential for pH regulation. *gcm2*, the gene mutated in *merovingian* mutants (Stawicki et al., 2014), encodes a transcription factor required for the generation of H^+^-ATPase rich ionocytes in zebrafish (Chang et al., 2009). Ionocytes are specialized cells in fresh water fish used to combat ion loss due to diffusion, and are believed to be the primary site of osmoregulation in these animals (Evans et al., 2005; Hwang and Lee, 2007). The H^+^-ATPase rich ionocytes that require *gcm2* are also enriched in the ${\text{Cl}}^{\text{−}}/{\text{HCO}}_{\text{3}}^{\text{−}}$ exchanger SLC4A1B (Lin et al., 2006; Lee et al., 2011), the gene mutated in the *persephone* mutant (Hailey et al., 2012). Knocking down either *gcm2* or *slc4a1b* results in decreased H^+^ excretion in zebrafish (Chang et al., 2009; Lee et al., 2011). The extracellular environment of hair cells both within the inner ear and lateral line is acidified in *gcm2* mutants (Stawicki et al., 2014). pH regulation has been shown to play a role in hearing. The H^+^-ATPase transporter and ${\text{Cl}}^{\text{−}}/{\text{HCO}}_{\text{3}}^{\text{−}}$ exchangers are enriched in the mammalian inner ear (Stanković et al., 1997; Everett et al., 1999; Lang et al., 2007). Additionally, mutations in subunits of the H^+^-ATPase transporter lead to sensorineural hearing loss in patients with distal renal tubular acidosis (Karet et al., 1999; Smith et al., 2000; Batlle and Haque, 2012). In agreement with the role of pH regulation in hearing, both of the pH regulating neomycin-resistant mutants identified showed decreases in FM1-43 uptake suggesting a decrease in mechanotransduction (MET) activity (Hailey et al., 2012; Stawicki et al., 2014). This decrease in MET activity is likely the mechanism of protection as both AG and cisplatin uptake have been shown to be dependent on MET activity (Gale et al., 2001; Marcotti et al., 2005; Alharazneh et al., 2011; Thomas et al., 2013), and both mutants show a decrease in uptake of fluorescently conjugated ototoxins (Hailey et al., 2012; Stawicki et al., 2014). While the role of these genes in MET activity limits their usefulness as targets to prevent ototoxicity, these findings highlight the ability of genetic screens to identify genes important for hair cell function in general as well as genes specifically modulating ototoxicity.

While forward genetic screens have proven useful in identifying novel genes involved in ototoxicity, with improved techniques in genome editing zebrafish will increasingly become a powerful system for testing the importance of candidate genes through reverse genetics. Groups have previously used zinc finger nucleases (ZFNs) and transcription activator-like effector nucleases (TALENs) to successfully generate targeted gene mutations in zebrafish (Doyon et al., 2008; Meng et al., 2008; Huang et al., 2011). More recently, groups have adapted the clustered regularly interspaced short palindromic repeats (CRISPR)-crispr associated protein (Cas) system for use in zebrafish (Chang et al., 2013; Hwang et al., 2013). Mutation rates using this system have been reported to be as high as 75–90% with an estimated rate of biallelic mutations at 56–81% (Jao et al., 2013), making screens in mutants in the F_0_ generation a possibility. This ability to rapidly generate mutants at a relative low cost make the zebrafish lateral line an ideal system for the initial testing of candidate genes and drug targets implicated in ototoxicity.

## Screening for compounds that modulate ototoxin-induced hair cell death

Many aspects of the zebrafish that make it useful for genetic screening also make it useful as a model for screening drug libraries and libraries of small drug-like molecules to identify modulators of hair cell toxicity. As lateral line hair cells are on the surface of the embryo most DMSO-solubilized compounds enter the hair cells when added to the surrounding media, allowing for easy drug delivery. Free-swimming larvae can be pretreated with compounds of interest, and then exposed to hair cell toxins like AGs or cisplatin. These exposures can be done in 96-well plates, so large libraries can be surveyed with redundant sampling to improve confidence in identified hits.

The Raible, Rubel, and Ou laboratories have screened a number of libraries looking for compounds that protect lateral line hair cells from either AGs or cisplatin. In 2008, we reported results from a screen of 10,960. We identified two compounds that robustly protect lateral line hair cells from neomycin exposure—both benzothiophene carboxamides (Owens et al., 2008). Since that initial report, we have screened other libraries, taking both unbiased and directed approaches, and have identified numerous protective compounds, a number of which are FDA approved drugs (Ou et al., 2009, 2012; Vlasits et al., 2012; Coffin et al., 2013). Hits from our unbiased screens are summarized in Table 1. While some hits in the screen protect against all ototoxins tested a number are specific to aminoglycosides (Table 1). This suggests that the two main classes of ototoxins kill hair cells through both overlapping and distinct pathways. It is generally believed that both classes of ototoxins act through ROS activation of cell death pathways (Schacht et al., 2012). In addition it has been shown that in zebrafish, MET activity is required for the uptake of both aminoglycosides and cisplatin (Wang and Steyger, 2009; Thomas et al., 2013). A further investigation into the cellular affects of drugs that either protect against both classes of ototoxins or one specific class of could provide new insight into how the uptake and toxicity of these compounds differ.

**Table 1:** **Otoprotective compounds identified through chemical screens**.

Protective drug	Protects against	Ototoxins tested	Function (if known)	Reference
Amodiaquine^$^	G, N	G, N	Histamine N-methyltransferase inhibitor	Ou et al. (2012)
Amsacrine^$^	G, N	G, N	Topoisomerase 2 poison	Ou et al. (2009, 2012)
Benzamil	C, G, K, N	C, G, K, N	Na^+^/Ca2^+^ channel blocker	Vlasits et al. (2012)
Carvedilol	N	N	Beta-2 adrenergic blocker	Ou et al. (2009)
Cepharanthine	N	N	Anti-inflammatory	Ou et al. (2009)
Chloroquine^$^	G, N	G, N	Inhibits SLC19A3	Ou et al. (2012)
Cinchonidine^$^	G, N	G, N	-	Ou et al. (2012)
Cinchonine^$^	G, N	G, N	-	Ou et al. (2012)
Drofenine	N	N	Acetylcholinesterase inhibitor	Ou et al. (2009)
Fluoxetine	G (st), N	C, G, K, N	Selective serotonin reuptake inhibitor	Vlasits et al. (2012)
Fluspirilene	G (st), N	C, G, K, N	Dopamine antagonist	Vlasits et al. (2012)
Hexamethyleneamiloride	N	N	Na^+^/H^+^ antiport inhibitor	Ou et al. (2009)
Loperamide	G, K, N	C, G, K, N	μ-opioid receptor agonist	Vlasits et al. (2012)
Mefloquine^$^	G, N	G, N	Inhibitor of histamine-N-methyl transferase	Ou et al. (2012)
Methiothepin	G, N	C, G, K, N	Serotonin and dopamine agonist	Vlasits et al. (2012)
Paroxetine	C, G, N	C, G, K, N	Selective serotonin reuptake inhibitor	Vlasits et al. (2012)
Phenoxybenzamine	G, N	C, G, K, N	Alpha-1 adrenergic blocker	Ou et al. (2009), Vlasits et al. (2012)
PROTO-1	N	C, N	-	Owens et al. (2008)
PROTO-2	N	N	-	Owens et al. (2008)
Quinine^$^	G, N	G, N	-	Ou et al. (2012)
Ractopamine	G, N, K	C, G, K, N	Beta-adrenergic agonist	Vlasits et al. (2012)
Raloxifene	G, N	C, G, K, N	Estrogen receptor modulator	Vlasits et al. (2012)
Tacrine	N	N	Acetylcholinesterase inhibitor	Ou et al. (2009)
Tamoxifen	G (st), N	C, G, K, N	Estrogen receptor modulator	Vlasits et al. (2012)

*C = Cisplatin, G = Gentamicin, K = Kanamycin, N = Neomycin, ST = short-term, ^$^ = Quinine ring derivative*.

A central issue for all of the hits identified in these screens is whether they affect the therapeutic activity of the drug of interest (e.g., the bactericidal activity of aminoglycosides). For the AGs, we can evaluate this by comparing inhibition of bacterial growth in the presence of the aminoglycoside with and without the protectant. Ideally, protective compounds will show no effect on the minimum bactericidal concentration (MBC) and minimum inhibitory concentration (MIC) needed to block bacterial growth. The majority of the compounds we have identified do not affect AG bactericidal activity, Benzamil being one notable exception (Owens et al., 2008; Ou et al., 2009; Vlasits et al., 2012).

Compounds that protect lateral line hair cells from aminoglycoside exposure span a wide range of targets, many of which were not anticipated. In several cases, we identified protective compounds that share characterized targets. One example is the family of selective estrogen receptor modulators (SERMs). Notably, while a number of these compounds protect hair cells from neomycin exposure (afimoxifene, MPP, raloxifene, tamoxifen, toremifene), others of this class known to affect estrogen receptors show no effect (Vlasits et al., 2012). In drug screening it is difficult to know whether the protective activity of a screen hit is due to known activities of the compound, or due to off target effects. However, because screening in the zebrafish is so efficient, we can quickly analyze groups of agonists or antagonists of specific pathways and processes to see if they share protective abilities.

Whether compounds that protect hair cells in zebrafish will protect hearing in mammals depends on an array of issues. FM1-43 can be used in zebrafish to rapidly test which compounds are affecting hair cell MET activity (Seiler and Nicolson, 1999), these compounds would have less utility in protecting mammalian hearing. The zebrafish system does not address whether compounds will reach appropriate targets in the mammalian inner ear, how the compounds will be metabolized, and whether they have toxicity issues, such as effects on the hERG (ether-a-gogo) channel in cardiomyocytes. The pharmaceutical industry has established tests for many of these issues. These first pass analyses address whether a compound is appropriate for continued research in a mammalian system. Due to the ease of screening in zebrafish one could design and test structural variants of a protective compound in hopes of maximizing both the compounds protective abilities as well as its pharmacological properties.

To test if compounds found in zebrafish screens protect mammalian hair cells we have used organotypic cultures of mature mammalian utricles (Warchol et al., 1993; Yamashita and Oesterle, 1995). Two compounds identified through zebrafish screens, PROTO and tacrine, have been shown to similarly protect hair cells in cultured mouse utricles (Owens et al., 2008; Ou et al., 2009). Culture system assays address whether protection is species specific. However, they do not address whether a protective compound will function in an intact animal. This is usually done in mammals and birds by repeated testing using the auditory brainstem response (ABR) method (Galambos and Hecox, 1978). ABRs can be repeatedly followed in individual animals over time to compare the normal response prior to an ototoxic agent with the response profile after exposure. This can be used to determine the effect of treatment with the putative protective drug at various dosages and times after treatments. These experiments are ongoing in the Rubel, Raible and Simon laboratories. While results have only been published to date in abstract form, preliminary results confirm the usefulness of zebrafish small molecule screening and dose finding comparisons as an effective platform to find molecules that can robustly modify hearing deficits due to AG exposure. In summary, a number of tools are now available to allow us to efficiently screen compounds in zebrafish and determine whether protective compounds may be appropriate for eventual clinical use to protect hearing.

## Live imaging of hair cell dynamics using the zebrafish lateral line

Understanding the route of ototoxin entry and intracellular trafficking may help explain why hair cells are particularly sensitive to ototoxic drugs like AGs and cisplatin. Such studies are greatly facilitated by the ability to conjugate fluorophores with ototoxins, yielding visible, traceable toxins that are structurally and functionally similar to the original molecule (Sandoval et al., 1998; Steyger et al., 2003). Versions of this method have been used to follow the trajectory AGs take following systemic injection. Such studies have shown that AGs rapidly cross the blood labyrinth barrier and accumulate apically within hair cells via the endolymph (Dai et al., 2006; Dai and Steyger, 2008; Wang and Steyger, 2009; Wang et al., 2010). The surface location of lateral line hair cells does not recapitulate this systemic trafficking; however, fluorescently conjugated AGs “administered” in embryo medium also accumulate within the apical domain of hair cells (Wang and Steyger, 2009). Their accumulation is dependent on functional mechanotransduction, as MET-deficient *myo7a* (*mariner*) and *cdh23* (*sputnik)* mutants, as well as larvae exposed to the pharmacological MET blockers quinine and amiloride, fail to accumulate fluorescently labeled AGs (Coffin et al., 2009; Wang and Steyger, 2009; Hailey et al., 2012; Ou et al., 2012). Similar results have been observed in mammals, both *in vivo* following systemic injection of fluorescent AG (Dai et al., 2006; Dai and Steyger, 2008; Wang et al., 2010) as well as in organotypic utriclular cultures (Alharazneh et al., 2011). Thus, mechanisms of AG entry appear to be conserved between zebrafish and mammals.

Using this tool to evaluate AG behavior, we can broadly categorize hits from our small molecule and genetic screens. The majority of characterized hits appear to protect lateral line hair cells from toxicity by preventing AG entry (Ou et al., 2009, 2012; Hailey et al., 2012; Vlasits et al., 2012; Stawicki et al., 2014). This is surprising, since these agents do not appear to modulate a shared cellular process or share a common drug target. That so many non-overlapping compounds and genes affect AG entry into lateral line hair cells suggest that MET properties are highly susceptible to even slight perturbation, even in seemingly unrelated intracellular pathways. We believe that results from these screens argue that potential otoprotectants be tested for their effects on toxin entry, lest they be ascribed an erroneous mechanism of action based upon their known targets. Given our results thus far, it is likely that a number of additional hits from future screens will confer protection from AGs, at least in part, by perturbing entry into hair cells.

While chemical and genetic screens performed by our labs and others take advantage of the ease with which one can assay hair cell number following toxin exposure, there are also fundamental aspects of hair cell biology and toxicity that are dynamic in nature and well-suited for zebrafish lateral line studies. Fluorescent biosensors engineered to monitor a variety of dynamic intracellular processes are constantly evolving, and the genetic malleability of the lateral line system enables rapid generation of hair cell lines expressing biosensors to uncover dynamic events central to ototoxin-induced hair cell death.

One dynamic process that is critical for hair cell function is intracellular Ca^2+^ homeostasis. Stimulation of MET elicits an intracellular Ca^2+^ response within hair cells (Ohmori, 1985; Ricci and Fettiplace, 1998; Ricci et al., 1998; Beurg et al., 2006, 2009, 2010), which contain a number of mobile Ca^2+^ buffers and extrusion mechanisms to tightly regulate cytoplasmic levels (Rabié et al., 1983; Baird et al., 1997; Steyger et al., 1997; Yamoah et al., 1998; Hackney et al., 2003, 2005). Longitudinal studies of AG toxicity in chick and mouse have suggested that intracellular Ca^2+^ is elevated during hair cell death (Hirose et al., 1999; Matsui et al., 2004). To provide a more complete picture of Ca^2+^ behavior during these events, we generated transgenic zebrafish larvae containing the Ca^2+^ biosensor GCaMP3 (Tian et al., 2009) within hair cells (Esterberg et al., 2013). We found that Ca^2+^ homeostasis is rapidly and dramatically disrupted within the cytoplasm following AG exposure in a manner that is completely predictive of cell death (Esterberg et al., 2013, 2014). This event follows an initial increase in mitochondrial activity and subsequent depolarization, and is consistent with mitochondrial overload phenotypes seen in AG toxicity (Ylikoski et al., 1974; De Groot et al., 1991; Lang and Liu, 1997; Dehne et al., 2002; Jensen-Smith et al., 2012). By targeting additional, and spectrally distinct, Ca^2+^ biosensors to the endoplasmic reticulum (ER) and mitochondria, the two largest intracellular Ca^2+^ stores, we pinpointed the origin of this disruption and uncovered a novel mechanism that appears to underlie AG-induced hair cell toxicity: Ca^2+^ is released from the ER in a manner that is highly correlative to an increase in mitochondrial Ca^2+^ (Esterberg et al., 2014). This is particularly compelling, as several studies have demonstrated that AGs bind directly to Ca^2+^ binding proteins and chaperones within in the ER (Horibe et al., 2004; Miyazaki et al., 2004; Karasawa et al., 2011). These data further suggest that a consequence of unregulated ER-mitochondrial Ca^2+^ transfer is mitochondrial dysfunction resulting in increase of ROS production within dying hair cells. While ROS is widely suggested as a causative factor of AG toxicity (Huth et al., 2011; Karasawa and Steyger, 2011; Schacht et al., 2012), our studies suggest that this is an obligatory event triggered by the loss of mitochondrial function. Thus, our work in the zebrafish has enabled us to identify novel AG toxicity mechanisms, and can potentially order them with other events known to regulate AG toxicity.

## Conclusion

The zebrafish lateral line system provides a useful platform with which to discover drugs, potential drugs, and genes that affect hearing and balance in the human population. Beyond their translational aspects, agents that promote hair cell survival possess the power to provide information about the pathways involved in these processes. A better understanding of events surrounding ototoxin-induced hair cell death will maximize our ability to predictively design drugs based on their target interactions.

## Conflict of interest statement

The authors declare that the research was conducted in the absence of any commercial or financial relationships that could be construed as a potential conflict of interest.
